# Crystal structures of archaeal 2-oxoacid:ferredoxin oxidoreductases from *Sulfolobus tokodaii*

**DOI:** 10.1038/srep33061

**Published:** 2016-09-13

**Authors:** Zhen Yan, Akane Maruyama, Takatoshi Arakawa, Shinya Fushinobu, Takayoshi Wakagi

**Affiliations:** 1 Department of Biotechnology, Graduate School of Agricultural and Life Sciences, The University of Tokyo, 1-1-1 Yayoi, Bunkyo-ku, Tokyo 113-8657, Japan

## Abstract

As the first three-dimensional structure of the two-subunit type 2-oxoacid:ferredoxin oxidoreductases (OFOR) from archaea, we solved the crystal structures of STK_23000/STK_22980 (StOFOR1) and STK_24350/STK_24330 (StOFOR2) from *Sulfolobus tokodaii*. They showed similar overall structures, consisting of two a- and b-subunit heterodimers containing thiamin pyrophosphate (TPP) cofactor and [4Fe-4S] cluster, but lack an intramolecular ferredoxin domain. Unlike other OFORs, StOFORs can utilize both pyruvate and 2-oxoglutarate, playing a key role in the central metabolism. In the structure of StOFOR2 in unreacted pyruvate complex form, carboxylate group of pyruvate is recognized by Arg344 and Thr257 from the a-subunit, which are conserved in pyruvate:ferredoxin oxidoreductase from *Desulfovbrio africanus* (DaPFOR). In the structure of StOFOR1 co-crystallized with 2-oxobutyrate, electron density corresponding to a 1-hydroxypropyl group (post-decarboxylation state) was observed at the thiazole ring of TPP. The binding pockets of the StOFORs surrounding the methyl or propyl group of the ligands are wider than that of DaPFOR. Mutational analyses indicated that several residues were responsible for the broad 2-oxoacid specificity of StOFORs. We also constructed a possible complex structural model by placing a Zn^2+^-containing dicluster ferredoxin of *S. tokodaii* into the large pocket of StOFOR2, providing insight into the electron transfer between the two redox proteins.

Oxidative decarboxylation of 2-oxoacids, such as pyruvate, 2-oxobutyrate and 2-oxoglutarate, is a key reaction of intermediary metabolism^1^. Most organisms exploit the reducing power of these substrates to reduce a low potential electron carrier and concomitantly form an energy-rich thioester linkage between CoA and the nascent acyl group. In mitochondria and many aerobic bacteria, the reactions are catalyzed by 2-oxoacid dehydrogenase in a multienzyme complex, which is composed of three components, namely E1 (binds to thiamin pyrophosphate, TPP), E2 (binds to lipoamide), and E3 (binds to flavins), with a molecular mass of 5–6 MDa^2^,^3^. In archaea, early branching bacteria, and amitochondrial eukaryotes, the reactions are catalyzed by a much simpler enzyme, 2-oxoacid:ferredoxin oxidoreductase (OFOR)^4^. The maximum total molecular mass of OFORs is approximately 270 kDa, but their subunit compositions vary depending on the organism: a_2_ type (homodimer), (ab)_2_ type (dimer of heterodimers), (abc)_2_ type (dimer of heterotrimers), (abcd)_2_ type (dimer of heterotetramers), or other types^5^,^6^. TPP, Mg^2+^ ion, and iron-sulfur cluster(s) are intrinsic cofactors. The external electron acceptor is usually ferredoxin (Fd), which converts from an oxidized to a reduced state in the oxidative reaction of 2-oxoacid^7^,^8^,^9^,^10^. Formation and breakdown of a stable free radical intermediate are the unique feature of the catalytic mechanism of OFOR^1^,^7^,^11^,^12^,^13^. The radical intermediate of OFOR was extensively characterized by electron paramagnetic resonance spectroscopy^14^.

A homodimeric (a_2_ type) pyruvate:ferredoxin oxidoreductase from *Desulfovbrio africanus* (DaPFOR) is the first OFOR whose crystal structures have been determined^15^,^16^. The large a-subunit (135 kDa) is composed of seven domains; TPP binds between domains I and VI, a proximal [4Fe-4S] cluster binds to domain VI, and median and distal [4Fe-4S] clusters bind to domain V (Fig. 1a,c). Domain V is also called the intramolecular Fd domain. The three [4Fe-4S] clusters are located between TPP and the protein surface, providing an electron pathway from pyruvate to an external electron acceptor (Fd protein) (Fig. 1c). A series of snapshot structures were obtained using this enzyme, and the structural changes in the active site from the unreacted pyruvate complex via a partially reacted tetrahedral intermediate to a stable TPP-based free radical form were visualized^17^,^18^.

Very recently, the second crystal structure of an enzyme in the OFOR family, oxalate oxidoreductase from *Moorella thermoacetica* (MtOOR), has been reported^19^,^20^. MtOOR contains a TPP and three [4Fe-4S] clusters, and its overall structure resembles DaPFOR (Fig. 1d). MtOOR is an (abc)_2_ type OFOR family member, and its a, b, and c subunits correspond to domains I–II, VI, and III–V, respectively (Fig. 1a). MtOOR oxidizes oxalate to form two molecules of CO_2_ and two low-potential electrons, but CoA is not required for the reaction.

We have been investigating the function of an (ab)_2_ type OFOR from the hyperthermophilic archaeon *Sulfolobus tokodaii* strain 7 (StOFOR1, Fig. 1a), which is composed of a 70 kDa a-subunit (STK_23000, corresponding to fusion domains III-I-II) and a 34 kDa b-subunit (STK_22980, corresponding to domain VI)^5^. StOFOR1 is one of the simplest versions of OFORs because it lacks the intramolecular Fd-like domain (domain V), providing a good model system for studying the molecular mechanism of this class of enzymes. A cognate 7Fe-containing dicluster type ferredoxin from the same organism (StFd, STK_01630)^21^ was shown to be an efficient electron acceptor, with an apparent *K*_m_ of 2–3 μM^22^,^23^. StOFOR1 shows broad substrate specificity toward 2-oxoacids, such as pyruvate, 2-oxobutyrate, and 2-oxoglutarate, suggesting that it has multiple roles in glycolysis, gluconeogenesis, the tricarboxylic acid cycle, and amino acid metabolism^5^. Mutational and affinity labeling studies of StOFOR1 identified the critical residues for substrate recognition and catalysis^24^,^25^,^26^. Recently, we proposed a comprehensive reaction pathway of StOFOR1 by mutating Cys residues, which are ligating the sole [4Fe-4S] cluster^27^. StOFOR1 forms a stable radical intermediate upon the addition of pyruvate, as commonly observed for other OFORs^28^,^29^. The [4Fe-4S] cluster in the a-subunit was required for the oxidative decarboxylation of pyruvate to yield acetyl-CoA, whereas the cluster and CoA were not required for the non-oxidative decarboxylation of pyruvate to yield acetaldehyde.

In this study, we report the crystal structures of StOFOR1 and its paralog StOFOR2 (STK_24350 and STK_24330), providing insights into the substrate recognition and reaction mechanism of the (ab)_2_ type OFORs from extremophilic archaea and bacteria.

## Results and Discussion

### Characterization of StOFOR2

**Figure 1:**
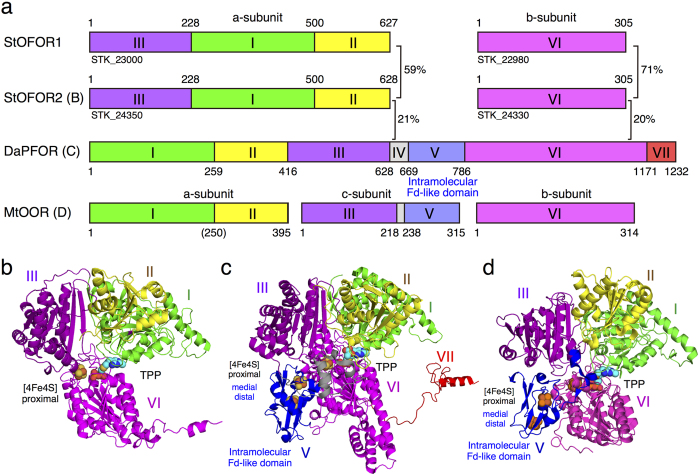
Schematics and similarities of the domains constituting the OFORs (**a**). Protomer structures for of StOFOR2 (**b**), DaPFOR (**c**) and MtOOR (**d**). The corresponding domains in StOFOR2, DaPFOR and MtOOR are shown in the same colors. TPP (cyan) and the [4Fe-4S] cluster (orange) are shown as spheres.

A paralogous gene set (*stk_24350*/*stk_24330*) of the StOFOR1 genes (*stk_23000*/*stk_22980*) is present in the *S. tokodaii* genome^30^. STK_24350 (a-subunit) and STK_24330 (b-subunit) have similar domain architectures to STK_23000 and STK_22980, with high amino acid sequence identity for both subunits (59% and 71%, Fig. 1a). Our previous study indicated that the OFOR activity in *S. tokodaii* cells is mainly due to StOFOR1^5^, and there is no evidence for the expression of the *stk_24350*/*stk_24330* genes. We prepared purified recombinant protein samples of STK_24350/STK_24330. The recombinant protein showed broad OFOR activities toward pyruvate and 2-oxoglutarate (Table 1); therefore, we designated this enzyme as StOFOR2. StOFOR2 exhibited a preference for pyruvate over 2-oxoglutarate. However, StOFOR2 has less activity compared with StOFOR1, due to its relatively higher *K*_m_ towards both substrates.

### Crystal structures of StOFOR1 and StOFOR2

The crystals of StOFOR1 grew in solutions containing PEG3350, and we could collect a 2.5 Å resolution data set (Table 2). However, the very low reproducibility of single crystal formation prevented phase determination and further crystallographic analyses. In contrast, StOFOR2 easily formed high-quality single crystals in solutions containing ammonium salts as the precipitant. We could solve the structure of StOFOR2 by the single-wavelength anomalous dispersion method using a selenomethionine(SeMet)-labeled protein crystal, and we determined the structures of the ligand-free (2.1 Å resolution) and pyruvate-complexed (2.2 Å) forms. The StOFOR1 structure was solved by molecular replacement using the StOFOR2 structure as a search model.

**Figure 2:**
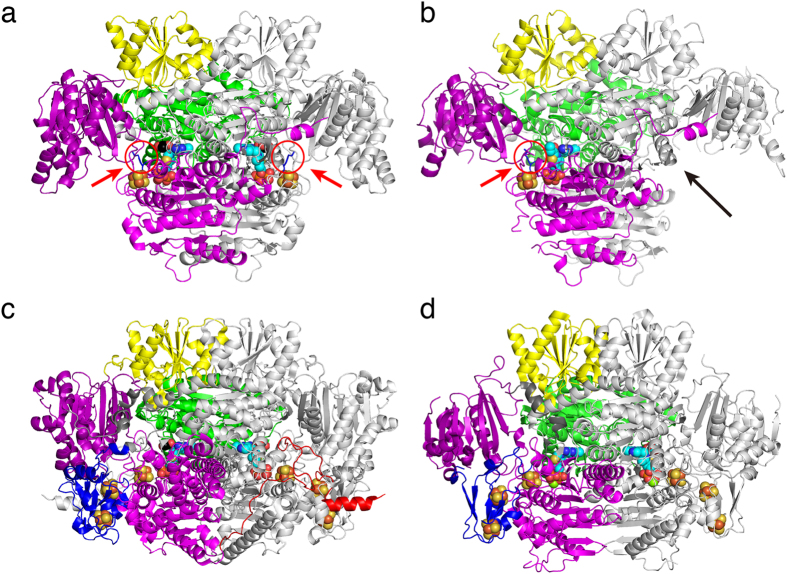
Overall structures of the OFORs in homodimer forms. (**a**) StOFOR2. (**b**) StOFOR1. (**c**) DaPFOR. (**d**) MtOOR. The domains are shown in the same colors as in Fig. 1, and the other protomer is shown in gray. TPP (cyan), the [4Fe-4S] cluster (orange), and pyruvate (black) are shown as spheres. The Lys125b residues (blue sticks) in StOFOR2 (**a**) and StOFOR2 (**b**) are indicated by red circles and arrows. A black arrow indicates the second protomer of StOFOR1, whose cofactors are lost.

**Figure 3:**
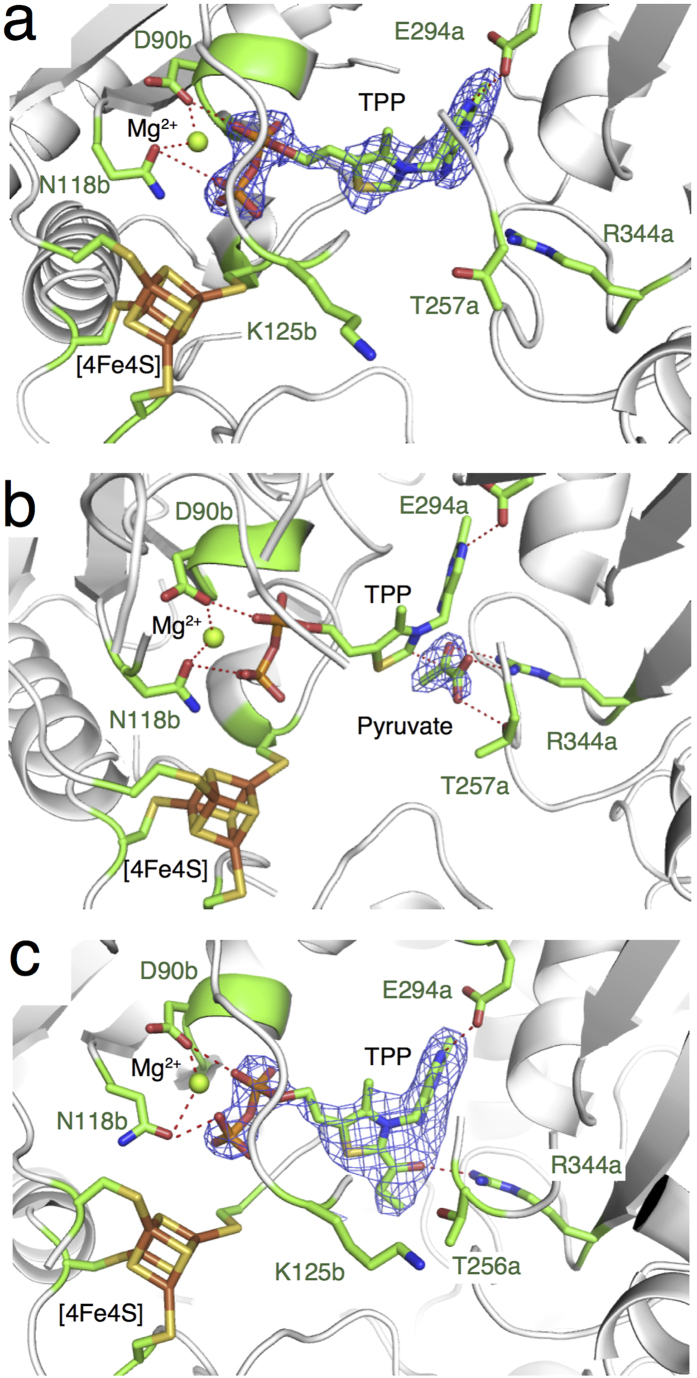
Active sites and |*F*_o_| − |*F*_c_| omit electron density maps (3.2σ) of StOFORs. (**a**) StOFOR2 in the substrate-free form. (**b**) StOFOR2 in complex with pyruvate. (**c**) StOFOR1 with a 1-hydroxypropyl TPP (post-decarboxylation state). TPP (cyan), Mg^2+^ (green), and the [4Fe-4S] cluster (orange) are shown as sticks and spheres.

StOFOR1 and StOFOR2 consist of two protomers (1 and 2), each of which consists of one heterodimer of a- and b-subunits. The asymmetric unit of StOFOR2 crystal contains one protomer, whereas that of StOFOR1 contains two protomers. StOFOR1 and StOFOR2 show high structural similarity (Root mean square deviation (RMSD) = 1.0 Å and number of aligned Cα atoms (Nalign) = 575 for a-subunit, and RMSD = 0.7 Å and Nalign = 286 for b-subunit). For StOFOR2, high-quality electron densities of the cofactors (TPP, Mg^2+^ ion, and [4Fe-4S] cluster) were observed in both protomers (Figs 2a and 3a; note that the two protomers of StOFR2 are crystallographically identical), whereas they were observed only in one protomer for StOFOR1 (Fig. 2b). Based on the |*F*_o_| − |*F*_c_| omit map in one protomer of StOFOR1, the TPP cofactor appeared to carry an additional group (Fig. 3c, discussed below). Some parts of the polypeptide chains in StOFOR1 could not be modeled due to low-quality electron density maps, presumably because of the oxidative destruction of the [Fe-S] cluster during crystallization. This may be one of the reasons for the low reproducibility of the StOFOR1 crystallization.

StOFOR2 and StOFOR1 possess domains III (1a–228a), I (228a–500a), and II (500a–627/628a) of the a-subunit and domain VI (1b–305b) of b-subunit but lack domains IV, V, and VII (Fig. 1a). The character (a or b) after the residue number indicates the subunit. The domain structures of StOFORs are basically similar to those of DaPFOR and MtOOR, regarding the spatial topology of domains I–III and VI (Fig. 1b–d). Domains I, II, and VI are mainly involved in the dimer interface of the two protomers in all OFORs (Fig. 2), whereas domains III and V are exposed on the protein surface. Domain VII in DaPFOR forms a long arm that extends over the other subunit^15^. In StOFORs, residues 229a–500a (domain I) and 501a–632a (domain II) are involved in the inter-subunit and inter-protomer interactions. Additionally, the C-terminal region of domain VI (283b–305b), which forms a short helix, extends over the other subunit and partly contributes to the dimer interface (Fig. 1b). This C-terminal extension of domain VI resembles domain VII in DaPFOR (Fig. 1c). Domain III of the a-subunit is stretched at both ends of the dimer, seemingly to maintain the balance of the enzyme as two wings. A database search using Dali server indicated that StOFOR2 is structurally similar to MtOOR (Z-score = 39.1, RMSD = 2.6 Å, and Nalign = 346 for a-subunit, and Z-score = 21.4, RMSD = 2.3 Å, and Nalign = 206 for b-subunit) and DaPFOR (Z-score = 37.6, RMSD = 2.7 Å, and Nalign = 346 for a-subunit, and Z-score = 19.6, RMSD = 2.6 Å, and Nalign = 222 for b-subunit).

The TPP molecules of StOFORs accompany a Mg^2+^ ion around their pyrophosphate group and are buried at the interface between the a-subunits and b-subunits. Based on the geometric consistency of the TPP binding site, we designated a pair of a- and b-subunits, which hold one TPP molecule, as one protomer (Fig. 2). Because StOFORs lack the intramolecular Fd-like domain V, there is a large pocket surrounded by domains III and VI in each protomer, which appears to be able to bind an external ferredoxin molecule (discussed below). In DaPFOR and MtOOR, the proximal, medial, and distal [4Fe-4S] clusters are arranged from TPP to the protein surface (Figs 1c,d and 2c,d). The medial and distal clusters are located in the intramolecular Fd domain V, whereas the proximal cluster is bound in the interior of domain VI. In StOFORs, the sole [4Fe-4S] cluster, which corresponds to the proximal cluster, is located near the surface of the large pocket (Fig. 2a,b). The two TPP cofactors in the functional (ab)_2_ heterotetramer are located at relatively close positions (16.5 Å for the nearest atoms), suggesting a possible structural influence between the two active sites. Kinetic and structural studies on various thiamine-dependent enzymes suggested a communication (alternating sites reactivity) between the cofactors in different protomers^31^,^32^,^33^.

### Cofactors and ligands

Despite the low sequence similarities with various other TPP-dependent enzymes, the invariant amino acids for TPP binding are also conserved in StOFORs. The Gly-Asp-Gly motif (89b–91b) and Asn118b are involved in binding the Mg^2+^ ion near the pyrophosphate group (Fig. 3). A conserved Glu294a is hydrogen-bonded to the N1′ atom of the 4-aminopyrimidine group, which plays an important role in the activation of TPP in enzymes (ylide formation)^34^,^35^,^36^,^37^,^38^.

The crystals of StOFOR1 were prepared by co-crystallization with 50 mM 2-oxobutyrate and 1 mM CoA. In the |*F*_o_| − |*F*_c_| omit map, we observed an additional group at the TPP thiazolium ring in protomer 1 (Fig. 3c). StOFOR1 forms a radical intermediate in the presence of pyruvate that is stable overnight, but it decays upon further addition of CoA^27^. The X-ray dose during the data collection was not extremely high (0.05 MGy as estimated by the Raddose-3D server)^39^, but it might have partly caused photoreduction of the crystal, possibly at the [Fe-S] clusters^40^. The crystallographic resolution of the StOFOR1 structure was not high enough to confidently determine the chemical structure of the additional group. However, the shape and size of the additional group are similar to a 1-hydroxypropyl group, which forms after decarboxylation of the covalently attached 2-oxobutyrate^27^. Therefore, for the crystallographic refinement model, we tentatively treated it as an enamine state of the 1-hydroxypropyl group by setting the stereochemical restrain of the bond between the 1-hydroxypropyl group and thiazolium ring as a double bond. The putative enamine state corresponds to the mono- and dihydroxyethyl-TPP intermediates observed in pyruvate oxidase^41^,^42^ and transketolase^43^, respectively. Because we could not exclude a possibility of presence of the radical intermediate at this site, we call it a post-decarboxylation intermediate (either enamine or free radical).

When the StOFOR2 crystal was soaked in a solution supplemented with 50 mM pyruvate for 30 min before flash cooling, we observed a separate density peak corresponding to the approximate size and shape of an unreacted pyruvate molecule (Fig. 3b). The TPP cofactor was almost invisible around the thiazolium moiety and exhibited a very high temperature factor (Table 2), probably because of the flexible nature of this group during the reaction^18^. The unreacted pyruvate can be well modeled in the |*F*_o_| − |*F*_c_| omit map, with a distance of 2.8 Å between the carbonyl carbon and the thiazolium ring of TPP, which can be designated as the pre-decarboxylated state.

### Active sites and substrate specificities

**Figure 4:**
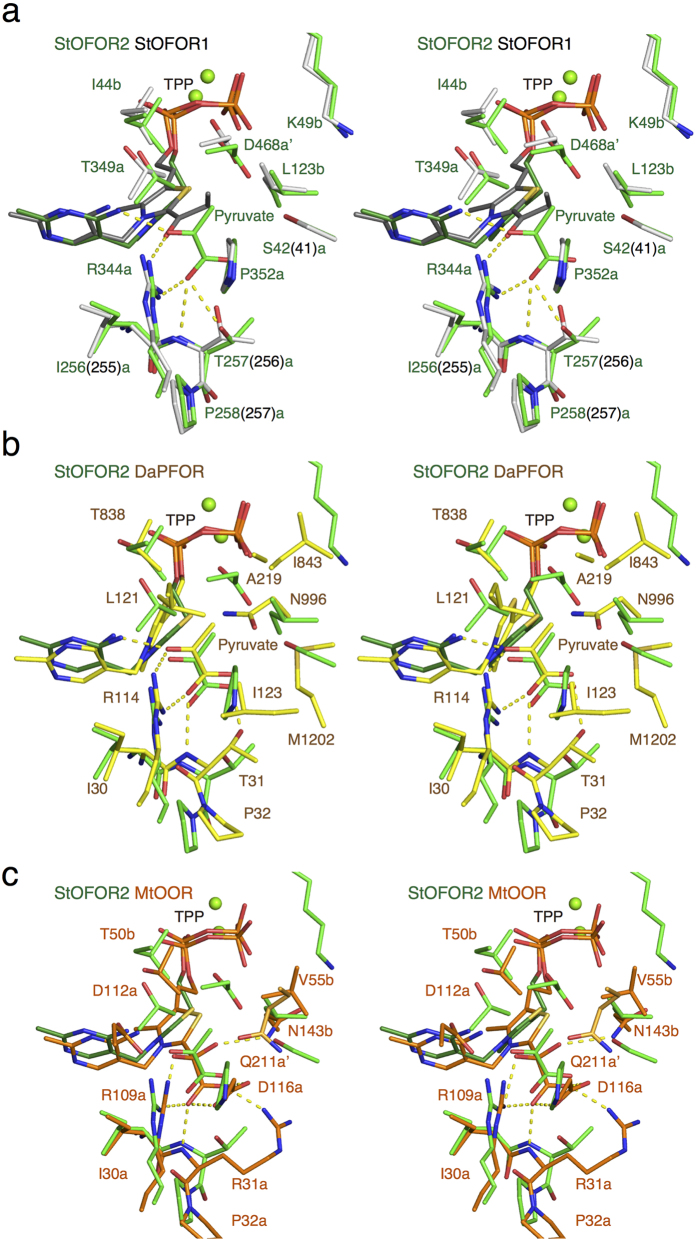
(**a**) Comparison of the 2-oxoacid substrate binding site of StOFOR2 and StOFOR1. StOFOR2 and the bound TPP are shown in green and dark green, and StOFOR1 and the bound 1-hydroxypropyl TPP (post-decarboxylation state) are shown in white and gray, respectively. The residue numbers of StOFOR2 are shown, and those of StOFOR1 are shown in parentheses, if they are different. (**b**) Comparison of StOFOR2 (green) and DaPFOR (yellow). The residue numbers of DaPFOR are shown. (**c**) Comparison of StOFOR2 (green) and StOFOR1 (orange). The residue numbers of MtOOR are shown.

Superimposition of the StOFOR1 and StOFOR2 structures indicates that they have almost identical active sites (Fig. 4a). Therefore, it is unclear why StOFOR2 shows higher *K*_m_ values for pyruvate and 2-oxoglutarate compared with StOFOR1 (Table 1). The carboxylate group of pyruvate is recognized by two highly conserved residues, Arg344a and Thr256a (Thr257a in StOFOR2). Several residues have slightly different numbers in StOFOR1 and StOFOR2, but we hereafter designate them with the numbers of StOFOR1. Arg344a forms bidentate hydrogen bonds with pyruvate. Thr256a consists of the YPITP motif of the OFOR family^24^, and the flanking residues (Ile255a and Pro257a) appear to be important for placing Thr256a in the correct position. Arg344a and all residues in the YPTIP motif in StOFOR1 play essential roles in the reaction because single mutants of these residues showed no activity or significantly decreased *k*_cat_ values^24^,^25^. The methyl group of pyruvate in StOFOR2 and the propyl group of 1-hydroxypropyl-TPP in StOFOR1 are surrounded by Ser41a, Thr349a, Asp468a’ (the prime mark indicates that the residue comes from the other protomer), Lys49b and Leu123b.

In clear contrast to StOFORs, DaPFOR is highly specific for pyruvate and unable to catalyze 2-oxoglutarate^10^. Superimpositions of the StOFR2 and DaPFOR structures illustrate that residues recognizing the carboxylate group of pyruvate (Thr31 and Arg114 in DaPFOR) are conserved, whereas those around the methyl group are not (Fig. 4b). DaPFOR has a narrower pocket that is surrounded by Leu121, Ala219, Ile843, Asn996, and Met1202. In particular, the side chain of Asn996 in DaPFOR significantly rotates upon pyruvate binding^18^. In StOFOR1, mutations at Lys49b and Leu123b, which correspond to Ile843 and Asn996 in DaPFOR, affected the substrate specificity^25^. The K49bR and L123bN mutants exhibited strong preferences for pyruvate and 2-oxoglutarate, respectively. However, no conformational change was observed in Leu123b of StOFOR2 upon pyruvate binding.

To further investigate the role of these residues in substrate specificity, four single mutants of StOFOR1 were constructed by mimicking the corresponding residues in DaPFOR (Table 1). S41aA and T349aL unexpectedly showed no significant difference from the wild type enzyme. In contrast, the D468aA and K49bI mutants lost their activity toward 2-oxoglutarate but retained their activity toward pyruvate, indicating that these residues play important roles in the catalysis of 2-oxoglutarate.

Figure 4c shows the superimposition of the StOFOR2 and MtOOR structures. The residue corresponding to Arg344a (Arg109a) is solely conserved, but the other residues are not. The positively charged Arg31a residue provides a binding site for the negatively charged substrate of MtOOR (oxalate). This residue constitutes a unique YPIRP motif in which the Thr residue of the YPITP motif is replaced with Arg. Arg31a and Asp116a play important roles in the unique “bait and switch” mechanism of MtOOR, introducing the substrate into the active site by the movement of a “plug loop”^20^. Therefore, StOFORs and MtOOR have largely different mechanisms for substrate recognition and incorporation.

### Modeling the structure of the StOFOR2 and StFd complex

**Figure 5:**
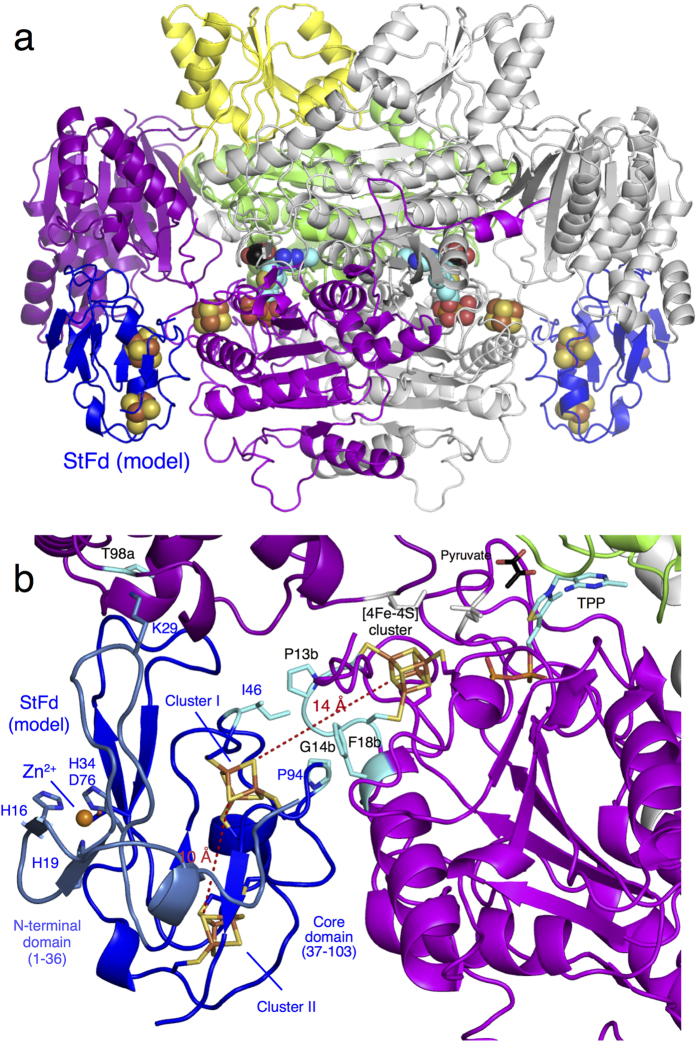
Structural model of the StOFOR2-StFd complex. (**a**) Structure of the complex with two ferredoxin molecules (StFd, blue) in the biological homodimer form. (**b**) Close-up view of the interactions and electron transfer pathway involving the TPP cofactor, [4Fe-4S] cluster in StOFOR2, and clusters I and II in StFd. The N-terminal Zn^2+^-binding domain of StFd is shown in light blue. A Zn^2+^ ion is shown as a red sphere. The residues involved in a possible interaction between StOFOR2 and StFd are shown in cyan.

The crystal structure of a cognate dicluster ferredoxin (StFd) from *S. tokodaii* has been determined^44^,^45^. We used the StOFOR2 structure to investigate its possible interactions with StFd because it has better quality than StOFOR1 at the molecular surface (Fig. 2a,b). StFd was modeled into the large pocket of StOFOR2 by aligning the two iron-sulfur clusters onto those of the Fd-like domain V of DaPFOR (Fig. 5a). StFd fitted well in the pocket to form a redox active complex. Two StFd molecules are held by the wing-like domains (domain III) of StOFOR2. A biochemical study indicated that StFd possess a [3Fe-4S] cluster (cluster I, −280 mV) and a lower potential [4Fe-4S] cluster (cluster II, −530 mV)^46^. In the crystal structure, cluster II lost the fourth iron atom, and both of the clusters were observed as [3Fe-4S] clusters^45^. The two clusters are properly superimposed with the medial and distal [4Fe-4S] clusters of DaPFOR and are geometrically adjacent to the sole [4Fe-4S] cluster of StOFOR2 (Fig. 5b). Cluster I is located 14 Å from the sole [4Fe-4S] cluster in StOFOR2, and the gap is filled with loops near the clusters. The sole [4Fe-4S] cluster in StOFOR1 has a redox potential of −545 mV^27^. Therefore, the potential differences, distances, and the pathway between the two clusters are appropriate for electron transfer. The redox potential of cluster II is much lower than that of cluster I; therefore, cluster II is thought to have lost its redox role, but it plays a structural role^46^. Cluster I of StFd also interacts with other enzymes or proteins, such as ferredoxin NADP^+^ oxidoreductase, which enables the redox recycling of Fd in *S. tokodaii*^47^.

Loops between cluster I in StFd and the sole cluster in StOFOR2 simultaneously provide a protein-protein interface and an electron transfer pathway (Fig. 5b). The loops from the two proteins mainly interact via hydrophobic residues, involving the side chains of Ile46 and Pro94 from StFd and Pro13b and Phe18b from StOFOR2. The loop in StOFOR2 (Cys12b–Cys15b) corresponds to a conserved CXGC motif, which ligates the proximal cluster of the OFOR family^19^. This loop is also completely conserved in StOFOR1 (data not shown).

StFd has a unique N-terminal Zn^2+^ binding domain (residues 1–36)^44^. However, its interactions with StOFOR2 are mainly mediated by the core domain (37–103) (Fig. 5b). A deletion study of the N-terminal domain showed that it is responsible for thermal stabilization but irrelevant to the catalytic activity of StOFOR1^22^,^23^. However, there are subtle interactions between the N-terminal domain of StFd and the wing-like domain III in StOFOR2. Lys29 of StFd may have been N6-monomethylated in a protein sample isolated from *S. tokodaii* cells^21^. The side chain of Lys29 was placed near the side chain of Thr98a in domain III, and it may add some interactions between the enzyme and the redox partner. Methylation of Lys is often reported in archaeal proteins, which may exert some effects on protein-protein interactions^48^. However, a tight electrostatic interaction has not been observed in the modeled complex structure. The protein-protein interactions can be accompanied by conformational changes^49^; therefore, the determination of the crystal structure of the StFd-StOFOR2 complex is required for a further detailed discussion.

### CoA binding site

To identify the CoA binding site, we tried to obtain the structure of the complex by soaking the crystals with different concentrations of CoA, acetyl-CoA, or their analogue, 4-fluoro-7-nitrobenzofurazan (NBD-F), for different incubation times. Unfortunately, a structure of the complex was not obtained. Moreover, the electron density map for the TPP cofactor was disordered, probably because of a reaction with CoA (data not shown). An affinity labeling study indicated that Lys125b of StOFOR1 is critical for the interaction with CoA^26^. Lys125a is conserved in both the StOFOR1 and StOFOR2 structures and is located in a pocket near the TPP cofactor (Fig. 2a,b). Binding of CoA to this putative site does not appear to interfere with Fd binding (Fig. 5a), suggesting that the formation of a ternary complex (StOFOR-Fd-CoA) is possible. The side chain of Lys125 penetrates into the cleft formed by the two TPP-binding domains (Fig. 3a,c). Therefore, decay of the hydroxyethyl-TPP radical intermediate after incubation with CoA can be explained^27^.

### Possible function of StOFOR2

In this study, we determined the crystal structures of two close OFOR paralogs of *S. tokodaii*. StOFOR1 has been extensively studied, while StOFOR2 has not yet been isolated from the cytosol of this microbe. However, the *stofor2* genes (*stk_24350* and *stk_24330*) do not seem to be pseudogenes because they contain a transcriptional promoter-like TATA box and a ribosome binding site in the upstream region. Therefore, they may be expressed *in vivo* under some culture conditions. For our biochemical and structural investigations, the *stofor2* genes had advantages because they were efficiently expressed in *E. coli* with full enzymatic activity and exhibited high crystallization reproducibility. We consequently determined the crystal structures of both StOFOR1 and StOFOR2, and their catalytic properties could be studied based on these structures.

### Comparison of the domain composition with OFOR family members

Compared with the structures of DaPFOR and MtOOR, StOFORs basically retain the same composition of the core domains (I, II, III, and VI) and the dimeric architecture with two identical protomers (Fig. 1). Despite the structural similarity, the sequence identity of each domain is very low between these enzymes (<22%). The conservation of the core structures among the distantly related OFOR family members with different domain and subunit compositions strongly supports the hypothesis that they have evolved from a common ancestor^5^,^6^.

Compared with DaPFOR, StOFORs do not have accessory domains (IV, V and VII). In particular, StOFORs lack an intramolecular Fd domain (V); therefore, their structures provided the simplest molecular architecture of the OFOR family enzymes. Our simple docking analysis showed that StOFORs can accommodate the cognate external Fd (StFd, Fig. 5) in a similar manner to the intramolecular Fd domains of DaPFOR and MtOOR (Fig. 2c,d). The intramolecular Fd domains of DaPFOR and MtOOR are different in their sizes; the former is 50% larger due to a 27-residue insertion^19^. The extended part of the intramolecular Fd domain in DaPFOR is exposed to solvent, and there is no obvious interaction with other domains^15^. This situation is similar to the N-terminal Zn^2+^-containing domain of StFd, which only shows a minor interaction with StOFOR2. The OFORs containing the intramolecular Fd domain can transfer electron(s) to the external Fd, but the detailed interaction between the enzyme and the redox partner remains unknown^16^.

Most OFOR family enzymes have three clusters, indicating that the domain composition contains an intramolecular Fd and is a major architecture of this class of enzymes^5^,^6^. However, several OFORs without an intramolecular Fd domain (*i*.*e*., containing a single [4Fe-4S] cluster) have been isolated from archaea and bacteria. Among them, pioneering studies on the reaction mechanism of this class of enzyme were accomplished using two (ab)_2_-type enzymes (pyruvate:ferredoxin oxidoreductase and 2-oxoglutarate:ferredoxin oxidoreductase) from the halophilic archaea *Halobacterium salinarum* (syn. *H. halobium*)^1^,^7^,^8^,^27^,^50^. In addition, OFORs from several hyperthermophilic and aerobic archaea and bacteria have a similar (ab)_2_-type subunit composition without an intramolecular Fd domain. For example, Ape2126/2128 and Ape1473/1472 from *Aeropyrum pernix*^51^ and 2-oxoglutarate:ferredoxin oxidoreductase (*korAB*) from *Hydrogenobacter thermophilus*^52^,^53^,^54^ have the same domain organization with StOFOR1 and StOFOR2. The crystal structures of StOFOR1 and StOFOR2 will provide the first structural basis for these “prototype” enzymes.

A structural comparison with DaPFOR, which shows specificity for pyruvate, and a mutational analysis of StOFOR1 revealed that Asp468a, Lys49b, and Leu123b were responsible for its wide substrate specificity, but mutations at Ser41a and Thr349a showed less of an effect (Table 1)^25^. KorAB from *H. thermophilus* shows low sequence homology to StOFOR1 and StOFOR2 (the sequence identities are approximately 30% for both the a- and b-subunits of both enzymes) but exhibits high specificity for 2-oxoglutarate^52^. Among the residues around the methyl group of pyruvate (Fig. 4a), the amino acids corresponding to Ser41a, Thr349a, Asp468a, and Leu123b of StOFOR1 are Ala, Thr, Leu, and Leu in KorAB. The N-terminal region of the b-subunit of KorAB is lost due to a 64-residue deletion; thus, a residue corresponding to Lys49b is absent. Therefore, the deletion of Lys49a and the replacement of Ser41a with Ala may increase the pocket size, and the replacement of Asp468a with Leu may reduce the negative charge to efficiently bind the carboxylate group of 2-oxogutarate.

### Biological implications

It is worth considering that the (ab)_2_ type OFORs without an intramolecular Fd domain are currently only observed in aerobic microbes living under extreme conditions, such as high temperature or high salt concentrations (*Sulfolobus*, *Aeropyrum*, *Halobacterium*, and *Hydrogenobacter*). Interestingly, these enzymes are relatively tolerant to oxygen, whereas OFORs are generally sensitive to oxidation^55^. It is not clear why StOFORs are tolerant to oxygen, but the four loops surrounding the sole [4Fe-4S] cluster (2b–17b, 117b–131b, 132b–152b, and 194b–210b, Fig. 5b) may have a protective role. For DaPFOR, an additional domain at the C-terminus covers the proximal [4Fe-4S] cluster, conferring unusual stability to oxygen^15^,^55^, which seems to improve the crystallization.

OFORs from anaerobic microorganisms are covalently inhibited by high concentrations of pyruvate (10–50 mM) under aerobic conditions^15^,^18^. The pyruvate inhibition of DaPFOR was shown to be caused by oxime formation with the N4′ of the aminopyrimidine ring^18^. In contrast, hyperthermophilic OFORs are structurally rigid and, therefore, may show specific features such as the lack of the pyruvate inhibition (Wakagi, unpublished data). This is probably due to the difficulty in forming a stable intermediate complex between OFOR and pyruvate, i.e., the long time required for the substrate to penetrate the active site at room temperature, etc.

## Methods

### Expression and purification of proteins

A pET-17b-based vector, pOFORAB^24^,^25^, was used to express StOFOR1. This vector was linearized using the following primer sets: *stk_23000 -* 5′-taaacctcaatggaattcgaag-3′ and 5′-catatgtatatctccttcttaaag-3′; and *stk_22980 -* 5′-taattttactcgagcagatccggc-3′ and 5′-catatgtatatctccttcgaattccattg-3′. The *stk_24350* and *stk_24330* genes encoding *StOFOR2* were amplified from the genomic DNA of *S. tokodaii* using the following primer sets: 5′-ggagatatacatatgactagaattgtttggatgatagg-3′ and 5′-ttccattgaggtttacgctccaccatacaatactacc-3′; and 5′-ggagatatacatatggtggagcgtaaacccgtatttg-3′ and 5′-gctcgagtaaaattaaattcttttggctttaataag-3′, respectively. According to the protocol of the in-fusion cloning kit (Takara Bio Inc., Kusatsu, Japan), the *stk_23000* and *stk_22980* genes in pOFORAB were replaced with *stk_24350* and *stk_24330*, respectively. *Escherichia coli* C43 (DE3) cells harboring the StOFOR1 or StOFOR2 expression plasmids were grown in Luria-Bertani medium containing 100 μg/ml ampicillin at 37 °C until the optical density at 600 nm reached 0.8–1.0. Then, 0.5 mM β-isopropylthiogalactoside, 0.1 mM FeSO_4_ and 30 μg/ml thiamin hydrochloride were added, and the cells were grown for an additional 20 h at 30 °C. The cells were then collected, suspended in 50 mM Tris-HCl (pH 8.0), and disrupted by sonication. The lysed cell suspension was heated at 80 °C for 20 min to denature the *E. coli* proteins. After centrifugation of the mixture to remove the denatured proteins, the supernatant was subjected to a DEAE Sepharose column (GE Healthcare, Fairfield, CT). The column was eluted with a linear gradient of NaCl (from 0 to 0.4 M) in 20 mM Tris-HCl (pH 7.5). Afterwards, the active fractions were subjected to Superdex-200 column chromatography (GE Healthcare); the column was equilibrated with 20 mM Tris-HCl (pH 7.5) and 250 mM NaCl. Selenomethionine (SeMet)-labeled protein was expressed using a methionine auxotrophic strain, *E. coli* B834 (DE3), and the cells were grown in Se-Met core medium (Wako Pure Chemical Ind., Osaka, Japan) supplemented with 10 g/L glucose, 250 mg/L MgSO_4_•7H_2_O, 4.2 mg/L FeSO_4_•7H_2_O, 10 ml/L KAO & Michaylak vitamin solution (Sigma-Aldrich, St. Louis, MO), 25 mg/L selenomethionine, and 100 μg/ml ampicillin. The procedures for expression and purification were identical to those of the native OFORs.

### Crystallography

All of the crystals were grown at 25 °C (StOFOR1) or 20 °C (StOFOR2) using sitting drop vapor diffusion. Drops were prepared by mixing a 1:1 volume of the protein and reservoir solutions. For crystallization of StOFOR1, the protein solution contained 7.2 mg/ml native protein, 50 mM sodium 2-oxobutyrate, 1 mM CoA (sodium salt hydrate) and 0.02 M spermine tetrahydrochloride, and the reservoir solution contained 15% PEG3350, 0.1 M Tris-HCl (pH 8.5) and 0.1 M NaBr. For crystallization of StOFOR2, the protein solution contained 15 mg/ml native or SeMet-labeled protein. The reservoir solution contained 0.7 M ammonium tartrate dibasic and 0.1 M Tris-HCl (pH 8.5) for native StOFOR2, and 4.0 M ammonium acetate and 0.1 M Bis-Tris-HCl (pH 7.0) for SeMet-labeled StOFOR2. The crystals were cryoprotected in the reservoir solutions supplemented with 25% PEG 400 (StOFOR1) or 25% glycerol (StOFOR2) and were flash-cooled at 100 K in a stream of nitrogen gas. The crystal of the complex was prepared by soaking the native StOFOR2 crystals in the cryoprotectant solution containing 50 mM sodium pyruvate. The X-ray diffraction data were collected at the Photon Factory, High Energy Accelerator Research Organization (KEK), Tsukuba, Japan. The diffraction data were processed using the HKL2000 program suite^56^. Initial phase calculations and model building of the StOFOR2 structure were performed using PHENIX^57^. The StOFOR1 structure was solved by molecular replacement using MOLREP^58^. Manual model building and structural refinements were performed using Coot^59^ and Refmac5^60^. Molecular graphics were prepared using PyMol (Schrödinger, LLC, New York, NY).

### Site-directed mutagenesis and enzyme assay

The T349L, S41A, D468A and K49I mutants of StOFOR1 were constructed using a PrimeSTAR Mutagenesis basal kit (Takara Bio Inc.) according to the manufacturer’s protocol. The following primers were used (the mutated sites are underlined): 5′-ccttcactaggtttacctactagaactg-3′ and 5′-taaacctagtgaaggacctcctctaat-3′ (T349L), 5′-tattacgctaatataaaaggaagacat-3′ and 5′-tatattagcgtaatactctctgtttcc-3′ (S41A), 5′-actggtgctgaacataatgaagagg-3′ and 5′-atgttcagcaccagtatagtacattg-3′ (D468A), and 5′-tctggtataataccgcatttctttag-3′ and 5′-cggtattataccagagcaaccaattc-3′ (K49I). All plasmids for the wild type and mutant enzymes were confirmed by DNA sequencing (Macrogen, Seoul, Korea).

The oxidative decarboxylation activities of the enzymes on pyruvate and 2-oxoglutarate were assayed based on the reduction of methylviologen, which was monitored by determining the absorbance at 578 nm at 80 °C as previously described^24^,^25^,^26^. The standard assay mixture (0.4 ml) included 50 mM Tris-HCl (pH 8.5), 10 mM pyruvate or 2-oxoglutarate, 0.25 mM CoA, 2 mM methylviologen, and the enzyme. Kinetic analysis was performed using 0.05–10 mM pyruvate or 0.1–50 mM 2-oxoglutarate.

## Additional Information

**Accession codes**: The structural ensemble has been deposited in the PDB under ID code 5B46, 5B47 and 5B48 for the native StOFOR2, the complex of StOFOR2 and pyruvate and StOFOR1, respectively.

**How to cite this article**: Yan, Z. *et al.* Crystal structures of archaeal 2-oxoacid:ferredoxin oxidoreductases from *Sulfolobus tokodaii. Sci. Rep.* **6**, 33061; doi: 10.1038/srep33061 (2016).

## Figures and Tables

**Table 1:** Kinetic parameters of StOFOR1, its mutants, and StOFOR2 for each substrate^a^.

	Pyruvate		2-Oxoglutarate	
	*K*_m_ (mM)	*V*_max_ (U/mg)	*K*_m_ (mM)	*V*_max_ (U/mg)
StOFOR1	0.32 ± 0.08	7.5 ± 1.0	2.1 ± 0.3	2.7 ± 0.4
StOFOR1 S41aA	0.49 ± 0.09	2.2 ± 0.2	3.2 ± 0.4	1.1 ± 0.1
StOFOR1 T349aL	0.51 ± 0.11	3.2 ± 0.4	2.3 ± 0.3	2.0 ± 0.2
StOFOR1 D468aA	—b	0.10 ± 0.03	—^b^	ND^c^
StOFOR1 K49bI	0.91 ± 0.12	2.1 ± 0.2	—b	ND^c^
StOFOR2	1.6 ± 0.3	7.0 ± 0.9	15 ± 2	1.4 ± 0.2

^a^0.25 mM CoA was used.

^b^Not determined.

^c^Not detected.

**Table 2:** X-ray data collection and refinement statistics.

	Se-Met StOFOR2	Native StOFOR2	StOFOR2 Pyruvate	StOFOR1
**Data collection**				
PDB entry		5B46	5B47	5B48
Beamline	NW12A	BL5A	NW12A	BL5A
Wavelength (Å)	0.9793	1.0000	1.0000	0.9780
Space group	*C222*_*1*_	*C222*_*1*_	*C222*_*1*_	*P2*_*1*_*2*_*1*_*2*_*1*_
Unit cell (Å)	*a* = 100.6*b* = 204.0*c* = 126.0	*a* = 101.9*b* = 205.0 = 127.0	*a* = 100.7*b* = 203.7*c* = 126.0	*a* = 75.2*b* = 145.9*c* = 170.1
Resolution (Å)^a^	50.00–2.50(2.54–2.50)	50.00–2.10(2.14–2.10)	50.00–2.20(1.99–2.20)	50.00–2.50(2.59–2.50)
Total reflections	674,899	574,608	478,719	281,054
Unique reflections	86,404	76,707	65,439	65171
Completeness (%)^a^	100.0 (100.0)	98.7 (97.9)	99.9 (100.0)	99.2 (93.7)
Redundancy^a^	7.8 (7.8)	7.5 (7.5)	7.3 (7.1)	4.3 (3.1)
Mean *I/σ* (*I*)^a^	24.3 (3.2)	30.3 (3.1)	32.1 (3.0)	27.0 (1.7)
CC 1/2	(0.932)	—b	(0.882)	—b
*R*_merge_ (%)^a^	8.9 (61.6)	7.3 (60.5)	6.2 (66.6)	5.8 (37.3)
**Refinement**				
Resolution (Å)		33.93–2.10	36.17–2.20	98.93–2.50
No. of reflections		72,834	61,011	61,283
*R*/*R*_free_ (%)		17.6/21.9	20.5/25.9	21.9/28.1
No. of atoms		7,592	7,310	13,074
Protein chains		A, B	A, B	A–D
Cofactors and ligands		TPP, Mg^2+^, [4Fe4S]	TPP, Mg^2+^, [4Fe4S]	1-hydroxy propyl-TPP, Mg^2+^, [4Fe4S] (A)
No. of solvents		263 (water)	191 (water)	61 (water)
**Average B-factor** (**Å**^**2**^)				
Protein atoms		42.2 (A + B)	53.1 (A + B)	67.7 (A + B)67.7 (C + D)
Cofactors and ligands		50.3 (TPP-Mg^2+^)39.5 ([4Fe-4S])	102 (TPP-Mg^2+^)68.5 ([4Fe-4S]) 76.9 (pyruvate)	58.0 (1-hydroxy-propyl-TPP-Mg^2+^) 60.8 ([4Fe-4S])
Waters		40.3	47.2	52.4
**RMSD from ideal values**				
Bond length (Å)		0.018	0.019	0.014
Bond angles (°)		1.93	1.96	1.76
**Ramachandran Plot** (**%**)				
Favored		97.3	96.1	93.8
Allowed		2.3	3.6	4.7
Outlier		0.4	0.3	1.5

^a^The numbers in parentheses indicate the highest resolution shell.

^b^The dataset was processed by HKL-2000 of versions earlier than 2.3.2.
